# *tilS* and *rpoB*: New Molecular Markers for Phylogenetic and Biodiversity Studies of the Genus *Thiothrix*

**DOI:** 10.3390/microorganisms11102521

**Published:** 2023-10-09

**Authors:** Nikolai V. Ravin, Dmitry D. Smolyakov, Nikita D. Markov, Alexey V. Beletsky, Andrey V. Mardanov, Tatyana S. Rudenko, Margarita Yu. Grabovich

**Affiliations:** 1 Institute of Bioengineering, Research Center of Biotechnology of the Russian Academy of Sciences, 119071 Moscow, Russia; nravin@biengi.ac.ru (N.V.R.); mortu@yandex.ru (A.V.B.); mardanov@biengi.ac.ru (A.V.M.); 2 Department of Biochemistry and Cell Physiology, Voronezh State University, Universitetskaya pl., 1, 394018 Voronezh, Russia; songolifreya@mail.ru (D.D.S.); nikita.markov257@mail.ru (N.D.M.); ipigun6292@gmail.com (T.S.R.)

**Keywords:** *Thiothrix*, genome, MAG, *tilS*, *rpoB*, set of bacterial core genes, ANI, phylogeny, colorless filamentous bacteria

## Abstract

Currently, the phylogeny of the genus *Thiothrix* is based on comparative whole genome analysis because of the high homology of the 16S ribosomal RNA gene sequences within the genus. We analyzed the possibility of using various conservative genes as phylogenetic markers for the genus *Thiothrix*. We found that the levels of similarity of the nucleotide sequences of the tRNA(Ile)-lysidine synthase (*tilS*) and the β subunit of RNA polymerase (*rpoB*) genes are in good agreement with the average nucleotide identity (ANI) values between the genomes of various representatives of the genus *Thiothrix*. The genomes of *Thiothrix* strains MK1, WS, DNT52, DNT53, and H33 were sequenced. Taxonomic analysis using both whole genomes and the *tilS* gene consistently showed that MK1 and WS belong to *Thiothrix lacustris*, while DNT52, DNT53, and H33 belong to *Thiothrix subterranea*. The *tilS* gene fragments were subjected to high-throughput sequencing to profile the *Thiothrix* mat of a sulfidic spring, which revealed the presence of known species of *Thiothrix* and new species-level phylotypes. Thus, the use of *tilS* and *rpoB* as phylogenetic markers will allow for rapid analyses of pure cultures and natural communities for the purpose of phylogenetic identification of representatives of the genus *Thiothrix*.

## 1. Introduction

The genus *Thiothrix* contains lithotrophs capable of oxidizing a range of reduced sulfur compounds. Bacteria lead an attached lifestyle and form abundant fouling both in natural biotopes with a constant supply of hydrogen sulfide and in anthropogenic wastewater treatment plant systems and phosphorus removal bioreactors [1].

A metagenomic analysis of the composition of microbial communities of hydrogen sulfide rich springs showed that the proportion of representatives of the genus *Thiothrix* can reach up to 40% of the total number of bacteria in the community [2]. This demonstrates that the genus *Thiothrix* has a significant impact on the fluxes of substances in their habitats.

The first attempts to describe bacteria from the genus *Thiothrix* were made by Rabenhorst as early as 1865 [3]. In 1888, Winogradsky named the new genus *Thiothrix* based on the results derived from studying the key features of the enriched culture [4]. However, due to the difficulty of cultivation, the first description of pure cultures began only with the isolation of a type species of the genus *Thiothrix* in 1983, *Thiothrix nivea* [5]. Currently, the genus *Thiothrix* belongs to the phylum *Pseudomonadota*, class *Gammaproteobacteria*, order *Thiotrichales*, family *Thiotrichaceae*. The genus *Thiothrix* is represented by eight validly described species and five MAG’s with Candidatus status [1]. We sequenced a number of complete genomes between 2020 and 2022, including strains from our own collection (*Thiothrix* sp. Ku-5, DNT52, DNT53, H33, MK1, WS, AS), strains from international microbial collections (*Thiothrix unzii* A1^T^, *Thiothrix fructosivorans* Q^T^, and *Thiothrix* sp. CT3), and three MAGs (A52, RT, and KT) [1,2,6] obtained from metagenomes of microbial fouling from geographically distant sulfide biotopes.

The methods of genomic systematics allowed us to identify *Thiothrix* strains AS, Ku-5, and CT3 as a new species of *Thiothrix litoralis* sp. nov. AS^T^, *Thiothrix subterranea* sp. nov. Ku-5^T^, and *Thiothrix winogradskyi* sp. nov. CT3^T^, respectively [2,6].

MAGs A52, RT, and KT, which were obtained by us, as well as MAGs *Thiothrix* sp. SSD2 and *Thiothrix* sp. 207, which are available in international databases, were identified based on an analysis of their full genomic data as five new species within the genus *Thiothrix*, *Candidatus* Thiothrix anitrata A52, *Candidatus* Thiothrix moscovensis RT, ‘*Candidatus* Thiothrix sulfatifontis’ KT, *Candidatus* Thiothrix singaporensis SSD2, and *Thiothrix* sp. 207, respectively [1,2,6]. Prior to our studies, none of the *Thiothrix* MAGs had been described as a new species—the isolates obtained belonged to already known species (*T*. *nivea*, *T*. *lacustris*) or had an uncertain taxonomic status at the species level due to problems in the taxonomy of members of the genus *Thiothrix*.

There was a problem in the taxonomic identification of isolates since most of them had a high level of 16S rRNA homology. In particular, the 16S rRNA homology between species of *T*. *fructosivorans*, *T*. *caldifontis* and *T*. *lacustris*, *T*. *litoralis*, *T. subterranea*, and *T. winogradskyi* varied between 98 and 100%, which placed them in one species according to the canonical similarity threshold of 97% [7]. This problem is complicated by the fact that representatives of the genus *Thiothrix* share a similar phenotype: filamentous colorless sulfur bacteria forming rosettes and intracellular inclusions of elemental sulfur under growth in the presence of hydrogen sulfide and thiosulfate, and as a rule, all representatives of the genus have a unified scheme of basic metabolic pathways [1].

Despite the development of new methods for taxonomic analysis, the study of the phylogeny of *Thiothrix* by 16S rRNA gene sequences is still difficult for a number of reasons. The 16S rRNA gene has long been the classical phylogenetic marker. To define the exact taxonomic position of *T*. *caldifontis* and *T*. *lacustris*, a number of housekeeping genes (*hsp60*, *gyrB*, *rpoD*, *rpoB*, *dnaJ*) were used for the first time, in addition to a polyphasic analysis of the obtained isolates, which allowed them to be confidently assigned to new species of the genus *Thiothrix* [8,9]. The lack of reliable phylogenetic markers made it difficult to determine the exact phylogenetic relationship of the already described species, as well as that of new isolates [10,11]. However, phylogeny based on the use of full genome sequences allowed for the clarification of the taxonomy of the genus *Thiothrix*. A full-fledged study of the species composition of *Thiothrix* from the natural community, using ANI analysis, involves obtaining the full genome sequences of pure cultures of representatives of the genus. However, this is difficult since the bacteria are hard to cultivate in laboratory conditions. This problem was partially solved with the advent of the ability to assemble MAGs from metagenomic sequences. However, the frequently observed presence of several similar but genetically different strains in an environmental sample prevents both the assembly of metagenomic contigs and their correct binning into MAGs.

The study of the ecological role of each species in biotopes is impossible without reliable phylogenetic markers. Currently, specific phylogenetic markers are being searched for in many bacterial groups [12,13,14]. In the present work, we analyzed several conserved genes and, based on the specificity of the genus *Thiothrix*, suggested a set of two genes, *tilS* and *rpoB*, the combination of which could establish the exact phylogenetic position of a species from biological sample. A universal primer system was developed to avoid the need for a assembly of full genome sequences to analyze the species composition of the biotope under study.

## 2. Materials and Methods

### 2.1. Sequencing and Analysis of tilS Gene Sequences

The total DNA was extracted from a microbial mat using Power Soil DNA isolation kit (MO BIO Laboratories, Inc., Carlsbad, CA, USA). PCR amplification of *tilS* gene fragments (619 bp) was carried out using primers TilS_F (cgcatcaYcagaaYgatcaggc) and TilS_R (tMtYccacaccaacacctgct). The PCR fragments were sequenced on Illumina MiSeq. Sequences starting from the TilS_F primer were clustered into operational taxonomic units (OTUs) at 97% nucleotide sequence identity using the USEARCH v.11 program [15]. Low-quality reads and chimeric sequences were removed by using the USEARCH algorithms. To calculate OTU abundances, all reads were mapped to OTU sequences at a 97% global identity threshold via the use of Usearch. The taxonomy of the obtained OTUs was analyzed using BLASTN searches against the NCBI non-redundant nucleotide sequence database. The phylogenetic tree was constructed based on the maximum likelihood [16,17]. The tree is drawn to scale, with branch lengths in the same units as those of the evolutionary distances used to infer the phylogenetic tree. The evolutionary distances were computed using the Kimura 2-parameter method and are in the units of the number of base substitutions per site. The numbers at the branch nodes are bootstrap values (expressed as percentages of 1000 replicates).

### 2.2. Sequencing and Assembly of Genomes of New Thiothrix Isolates

Genomic DNA was isolated using a DNeasy PowerSoil DNA isolation kit (Mo Bio Laboratories, Carlsbad, CA, USA) and sequenced using Illumina and/or Oxford Nanopore technologies. For the Illumina sequencing of the DNT52, MK1, WS and H33 strains, the shotgun genome library was prepared using the NEBNext Ultra II DNA library prep kit (New England Biolabs, Ipswich, MA, USA) and sequenced on an MiSeq instrument in a paired reads mode (2 × 300 nt). About 10 Gbp of Illumina reads was generated for each strain. Low-quality sequences were trimmed using Sickle v.1.33 (q = 30).

The genome of *Thiothrix* sp. H33 was assembled using SPAdes v.3.13.0 [18]. The total length of the contigs was 4,297,497 bp, with a N50 contig size of 124,522 bp.

The genomic DNA of the DNT52, MK1, and WS strains were additionally sequenced on a MinION device (Oxford Nanopore Technologies, Oxford, UK) using the ligation sequencing kit 1D and FLOMIN110 cells. A total of 130,791 (939 Mbp), 54,804 (383 Mbp), and 147,323 (1347 Mbp) reads were obtained for the DNT52, MK1, WS strains, respectively. For the DNT52 and WS strains, MinION reads were assembled into contigs using Flye v.2.8.2 [19]. The consensus sequence of the assembled contigs was corrected with two iterations of Pilon v.1.22 [20] using the Illumina reads. Flye v.2.8.2 and SPAdes v.3.13.0 were used to assemble the *Thiothrix* sp. MK1 genome.

*Thiothrix* sp. DNT53 genome sequencing on MinION generated 163,542 reads with a total length of 1.2 Gbp. These reads were assembled using Flye v.2.8.2.

### 2.3. Genomic Sequence Analysis

For genome-based phylogenetic analysis, GTDB-Tk v.2.1.1 [21] was used to identify 120 single copy marker genes in the genomes and to create a multiple sequence alignment of concatenated genes sequences. A maximum likelihood tree was estimated from the alignment by PhyML v.3.3 [22] using default parameters (LG amino acid substitution model, four substitution rates categories modeled by discrete gamma distribution with estimated shape parameters; branch support values were calculated via the approximate Bayes method.)

ANI was calculated using an online resource (https://www.ezbiocloud.net/tools/ani (accessed on 28 April 2023)) based on the OrthoANIu algorithm using USEARCH [23].

The selection of degenerate primers was performed using the Primer-BLAST tool (https://www.ncbi.nlm.nih.gov/tools/primerblast/index.cgi?LINK_LOC=BlastHome (accessed on 15 January 2023)).

## 3. Results and Discussion

### 3.1. Genome Sequences of New Thiothrix Isolates

The genome of *Thiothrix* sp. DNT52 was assembled into two circular contigs: 4,255,904 bp long chromosome and 151,453 bp long plasmid. One linear 3,955,684 bp long contig and two circular plasmid contigs with lengths of 141,598 bp and 20,159 bp. were obtained for *Thiothrix* sp. WS. The *Thiothrix* sp. MK1 genome was assembled into three contigs: one circular 3,612,515 bp long chromosome and two circular plasmids (21,587 bp and 13,540 bp). The *Thiothrix* sp. DNT53 genome was obtained as a single 4,204,657 bp-long circular chromosome. The draft genome of *Thiothrix* sp. H33 was assembled into 124 contigs with a total length of 4,297,497 bp.

The main characteristics of the obtained genomes are shown in Table 1.

### 3.2. The Invalidity of the Classical Phylogenetic 16S rRNA Marker

Doubts about the reliability of 16S rRNA gene as an informative phylogenetic marker appeared after the study of a number of bacterial groups (*Acidipropionibacterium*, *Cutibacterium*, *Propionibacterium*, *Pseudopropionibacterium*, *Lactobacillales*, *Rhodococcus*, etc.) for which significant phenotypic differences were observed [12,13,14], while 16S rRNA homology gave an unclear taxonomic view. The use of full genome sequence analysis methods (ANI, dDDH, AAI indices) showed that 16S rRNA was imperfect as a molecular marker for a number of taxonomic groups of prokaryotes.

**Figure 1:**
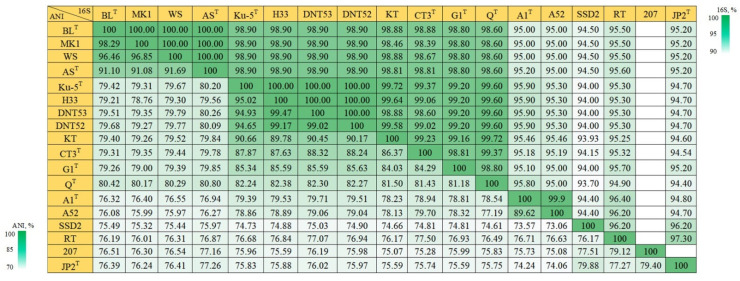
Heatmap comparing 16S rRNA gene homology and ANI scores for the genus *Thiothrix*: *T*. *lacustris* BL^T^ (GCF_000621325.1); *T*. *lacustris* MK1 (CP133218); *T*. *lacustris* WS (CP133333); *T*. *litoralis* AS^T^ (GCF_017901135.1); *T*. *subterranea* Ku-5^T^ (GCF_016772315.1); *T*. *subterranea* H33 (JAVFKN000000000); *T*. *subterranea* DNT53 (CP133197); *T*. *subterranea* DNT52 (CP133216); ‘*Ca*. Thiothrix sulfatifontis’ KT (GCA_022828425.1); *T*. *winogradskyi* CT3^T^ (GCA_021650935.1); *T*. *caldifontis* G1^T^ (GCF_900107695.1); *T*. *fructosivorans* Q^T^ (GCA_017349355.1); *T*. *unzii* A1^T^ (GCA_017901175.1); *Ca*. Thiothrix anitrata A52 (GCF_017901155.1); *Ca*. Thiothrix singaporensis SSD2 (GCA_013693955.1); *Ca*. Thiothrix moscovensis RT (GCA_016292235.1); *Thiothrix* sp. 207 (GCA_018813855.1); *T*. *nivea* JP2^T^ (GCF_000260135.1).

For the genus *Thiothrix*, the failure of the 16S rRNA gene as a phylogenetic marker has been repeatedly shown in a number of studies [2,7,10,11]. Of the species validly described as of 2018, only *T*. *nivea* and *T*. *unzii* were found to have 16S rRNA homology, with other *Thiothrix* species at 94.40–95.20% and 94.40–95.80, respectively (Figure 1), which meets the Yarza’s criterion for species separation (less than 97% similarity) [24]. For species of *T*. *lacustris*, *T*. *caldifontis*, and *T*. *fructosivorans*, homology values ranged from 98.6 to 98.8%.

The pair of *T*. *lacustris* BL^T^ and *T*. *litoralis* AS^T^ is a good example. At first, based only on the 16S rRNA gene sequence homology, which is 100% for this pair, the AS strain was assigned to the *T*. *lacustris* species. However, after obtaining the genomic sequence of strain AS, it was found that the ANI between the genomes of *T*. *lacustris* BL^T^ and strain AS is 92%, i.e., lower than the threshold needed for species separation (95%) [25,26]. This indicates that the AS strain belongs to another species named *Thiothrix litoralis* AS^T^ (Figure 1) [6].

The *Thiothrix* sp. CT3 has not been assigned to any *Thiothrix* species for 30 years due to the high sequence identity of the 16S rRNA gene sequence (98.8–99.4%) and the phenotypically and phylogenetically similar species *T*. *fructosivorans* Q^T^, *T*. *caldifontis* G1^T^ and *T*. *lacustris* BL^T^ [11]. However, phylogenetic analysis based on the complete genome sequence showed that this strain represents a distinct species, *T*. *winogradskyi* CT3^T^ [2].

A similar situation was also observed for the new species Ca. Thiothrix anitrata A52, which showed 99.9% 16S rRNA sequence homology with *T*. *unzii* A1^T^; the ANI between these genomes is 89.62%, which allows us to identify it as a separate species within the genus (Figure 1) [6].

At present, phylogenetic analysis based only on the 16S rRNA sequence homology does not allow for nine representatives of the genus to be unambiguously assigned to one of the species due to the high identity of 16S rRNA genes in this group (>96%).

### 3.3. The Choice of Phylogenetic Markers

The search for potential phylogenetic markers was performed using a set of bacterial core genes [27,28]. More than one hundred putative phylogenetic markers proved unsuitable for analyzing the systematics of the genus *Thiothrix*, primarily because of the high level of gene homology between different species (>98%).

Based on the analysis of standard phylogenetic markers for closely related bacteria, we evaluated the most frequently used genes: *dnaX*—DNA polymerase III subunit gamma; *gapA*—glyceraldehyde-3-phosphate dehydrogenase subunit A; *glnS*—glutamine-tRNA ligase; *gyrB*—DNA gyrase subunit B; *gyrA*—DNA gyrase subunit A; *hisA*—phosphoribosylformimino-5-aminoimidazole carboxamide ribotide isomerase; *recN*—DNA repair protein; *infB*—translation initiation factor 2; *hsp60*—heat shock protein gene; *recA*—recombinase A; *fusA*—elongation factor G; *rpoD*—DNA-directed RNA polymerase subunit D; *rspB*—L-gulonate 5-dehydrogenase; *infC*—translation initiation factor IF-3. These genes also appeared to be inappropriate for the taxonomy of the genus *Thiothrix* because of the high level of gene homology between strains of the same species, as well as the closely related representatives, or the mismatch between the topology of the obtained trees and the tree based on 120 conserved proteins.

### 3.4. An Alternative Set of Phylogenetic Markers

**Figure 2:**
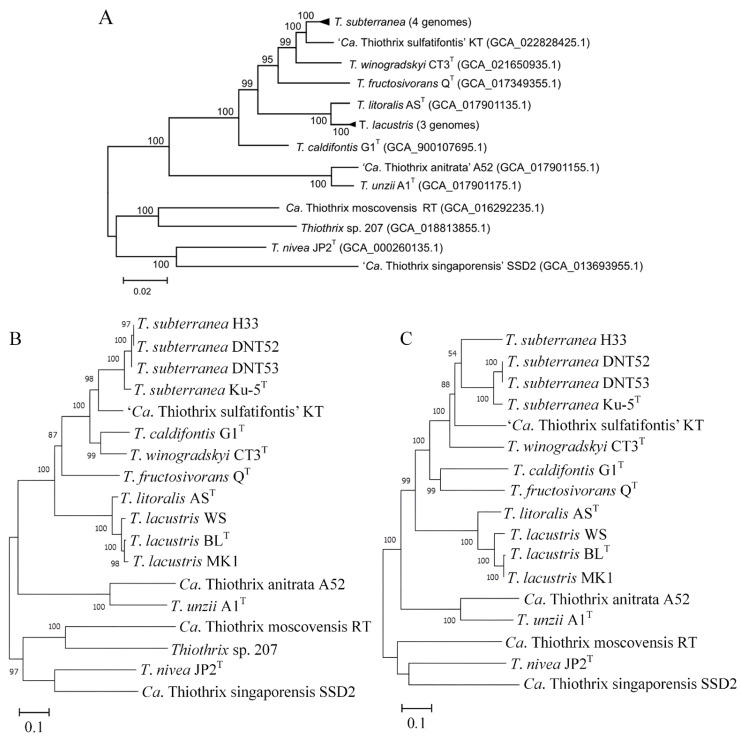
(**A**) The maximum likelihood tree of the genus *Thiothrix* based on the concatenated sequences of the 120 conserved marker genes. The GenBank assembly accession numbers are listed after the genome names. The internal branching support levels assessed by the Bayesian test in PhyML are specified at nodes. (**B**,**C**) The maximum likelihood trees of the genus *Thiothrix* based on the complete nucleotide sequences of the *tilS* (**B**) and *rpoB* (**C**) genes.

Based on the obtained data, after comparing tree topology and the homology level of the marker genes tested in this work, only two genes (*rpoB* and *tilS*) maximally matched with the phylogenetic tree topology based on the analysis of the concatenated sequences of the 120 conserved marker proteins (Figure 2).

### 3.5. The tilS Gene as a New Phylogenetic Marker for the Genus Thiothrix

The product of the *tilS* gene modifies lysine to a specific lysidine product, which in turn changes the specificity of the tRNA(Ile) codon from AUG to AUA [29]. This gene was found only in bacteria.

For *tilS*, two gene regions were evaluated: from 420 to 1003 nt and a shorter region from 596 to 956 nt. In a pairwise comparison of the *tilS* gene regions within the genus *Thiothrix*, it was found that the homology between the gene region and between the whole gene differs only by 1–3%. In turn, the homology of both the gene regions and the whole gene sequences differs by 1–2% from genome-wide ANI values, which allowed us to extrapolate the homology of the *tilS* gene to the ANI value to determine whether strains belong to the same or different species. Consequently, sequencing of the *tilS* gene region made it possible to predict the ANI value with an extremely small error. This approach excludes the need for whole genome sequencing.

**Figure 3:**
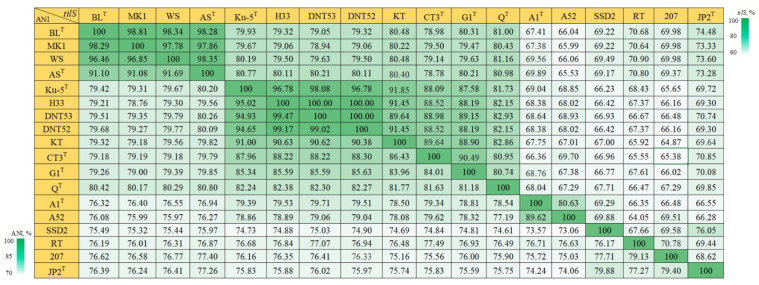
Heatmap comparing *tilS* gene homology and ANI scores for the genus *Thiothrix*: *T*. *lacustris* BL^T^ (GCF_000621325.1); *T*. *lacustris* MK1 (CP133218); *T*. *lacustris* WS (CP133333); *T*. *litoralis* AS^T^ (GCF_017901135.1); *T*. *subterranea* Ku-5^T^ (GCF_016772315.1); *T*. *subterranea* H33 (JAVFKN000000000); *T*. *subterranea* DNT53 (CP133197); *T*. *subterranea* DNT52 (CP133216); ‘*Ca*. Thiothrix sulfatifontis’ KT (GCA_022828425.1); *T*. *winogradskyi* CT3^T^ (GCA_021650935.1); *T*. *caldifontis* G1^T^ (GCF_900107695.1); *T*. *fructosivorans* Q^T^ (GCA_017349355.1); *T*. *unzii* A1^T^ (GCA_017901175.1); *Ca*. Thiothrix anitrata A52 (GCF_017901155.1); *Ca*. Thiothrix singaporensis SSD2 (GCA_013693955.1); *Ca*. Thiothrix moscovensis RT (GCA_016292235.1); *Thiothrix* sp. 207 (GCA_018813855.1); *T*. *nivea* JP2^T^ (GCF_000260135.1).

However, the *tilS* gene, as well as its regions, proved unsuitable in establishing phylogenetic relatedness between *T*. *lacustris* BL^T^ and *T*. *litoralis* AS^T^, as the *tilS* sequence similarity between them was 98.28% (Figure 3). Comparing the *tilS* gene of *T*. *litoralis* AS^T^ with strains MK1 and WS of *T*. *lacustris* showed a homology above 98%, which indicates that strain AS belongs to *T*. *lacustris*. Before that, *T*. *litoralis* AS^T^ was classified as a species of *T*. *lacustris* based on 16S rRNA analysis (100% homology), but obtaining the full genome sequence allowed it to be described as a separate species due to the 92% ANI between them (Figure 3).

The evaluation of the *tilS* gene as a universal phylogenetic marker was carried out on *T*. *subterranea* Ku-5^T^ and a group of strains: DNT52, DNT53, H33. The ANI values between these three strains and the species *T*. *subterranea* Ku-5^T^ were about 95%, indicating that they likely belong to *T*. *subterranea*. The *tilS* homology for *T*. *subterranea* Ku-5^T^ and the group of strains, DNT52, DNT53, and H33, was in the range of 96.78 to 98.08%. These data are also valid for the investigated *tilS* gene regions.

Thus, the *tilS* gene can be used to determine phylogenetic relatedness within the genus *Thiothrix*; however, as an exception, it proved to be inapplicable for the separation of *T*. *lacustris* and *T*. *litoralis*.

### 3.6. The rpoB Gene as an Alternative Phylogenetic Marker for the Genus Thiothrix

**Figure 4:**
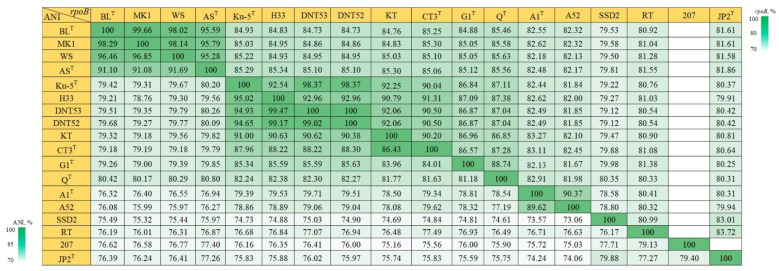
Heatmap comparing *rpoB* gene homology and ANI scores for the genus *Thiothrix*: *T*. *lacustris* BL^T^ (GCF_000621325.1); *T*. *lacustris* MK1 (CP133218); *T*. *lacustris* WS (CP133333); *T*. *litoralis* AS^T^ (GCF_017901135.1); *T*. *subterranea* Ku-5^T^ (GCF_016772315.1); *T*. *subterranea* H33 (JAVFKN000000000); *T*. *subterranea* DNT53 (CP133197); *T*. *subterranea* DNT52 (CP133216); ‘*Ca*. Thiothrix sulfatifontis’ KT (GCA_022828425.1); *T*. *winogradskyi* CT3^T^ (GCA_021650935.1); *T*. *caldifontis* G1^T^ (GCF_900107695.1); *T*. *fructosivorans* Q^T^ (GCA_017349355.1); *T*. *unzii* A1^T^ (GCA_017901175.1); *Ca*. Thiothrix anitrata A52 (GCF_017901155.1); *Ca*. Thiothrix singaporensis SSD2 (GCA_013693955.1); *Ca*. Thiothrix moscovensis RT (GCA_016292235.1); *Thiothrix* sp. 207 (GCA_018813855.1); *T*. *nivea* JP2^T^ (GCF_000260135.1).

The sequence of the *rpoB* (the beta subunit of RNA polymerase) gene was also analyzed as a phylogenetic marker. The topology of the phylogenetic tree based on *rpoB* nucleotide sequences and the tree based on 120 conserved proteins nearly matched. Also, unlike *tilS*, the necessary resolution was found to separate *T*. *lacustris* and *T*. *litoralis*. The *rpoB* homology values between *T*. *lacustris* BL^T^ and strains MK1 and WS of the same species are 99.7 and 98.0%, and the *rpoB* homology values between these three *T*. *lacustris* strains and the type strain *T*. *litoralis* AS^T^ are 95.3–95.8%. The *rpoB* homology values for *T*. *subterranea* Ku-5^T^ and the group of strains DNT-52, DNT-53, H33 are 98.3–98.4% (Figure 4).

Our analysis of the *rpoB* gene revealed a region with higher resolution at position 481–836 nt. The investigated region reliably separated the pair of *T*. *lacustris* BL^T^ and *T*. *litoralis* AS^T^ (Figure S1). The homology between the *T*. *lacustris* BL^T^ and *T*. *litoralis* AS^T^ in this region of *rpoB* was 91%, whereas between the *T*. *lacustris* strains, it ranged from 97.2 to 100%; for *T*. *subterranea* Ku-5^T^ and strains DNT-52, DNT-53, and H33, it ranged from 98 to 98.3%.

### 3.7. Application of the tilS Gene to Analyze the Diversity of Bacteria of the Genus Thiothrix in a Microbial Mat

Previously, we analyzed the microbial community of a bacterial mat from a hydrogen sulfide drainage well of the flooded Severnaya mine in Kemerovo, Russia, by subjecting the 16S rRNA gene fragments to high-throughput sequencing [30]. However, all obtained sequences belonging to *Thiothrix* had more than 97% similarity with several previously described species, which did not allow us to assess the species diversity of *Thiothrix*.

**Figure 5:**
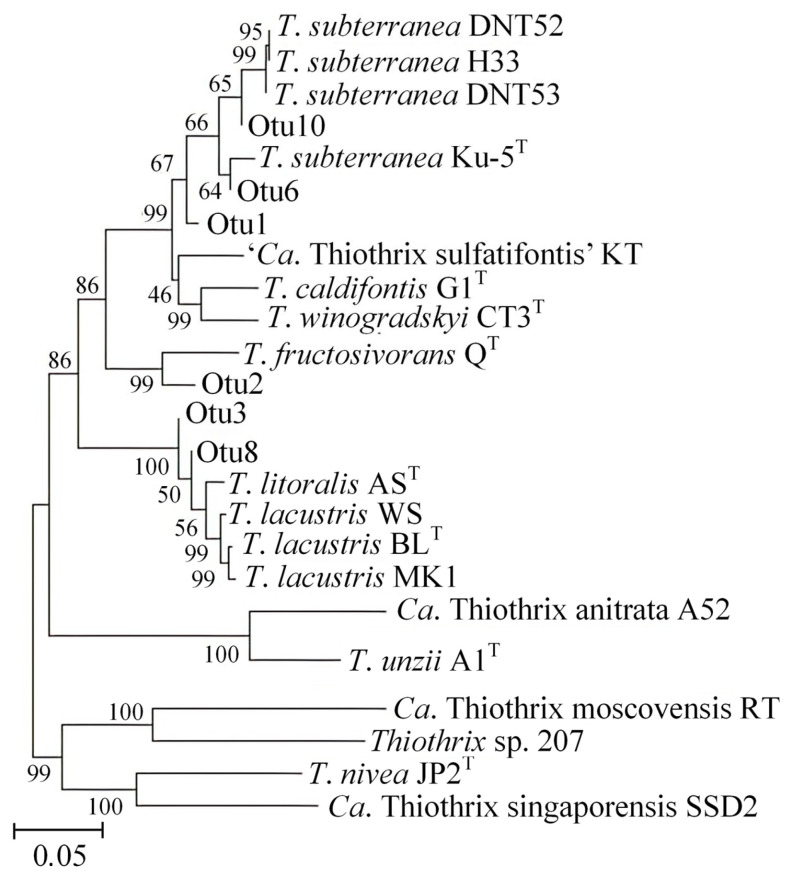
The maximum likelihood tree of the genus *Thiothrix* based on the sequences of the *tilS* gene. The internal branching support levels (assessed by the Bayesian test in PhyML) are specified at nodes.

Therefore, we profiled the composition of the microbial mat via the sequencing of the *tilS* gene fragments. Clustering of the 14,750 sequences obtained resulted in the identification of six operational taxonomic units OTUs of the genus *Thiothrix*. The phylogenetic tree showed that the obtained OTUs clustered with already described species and formed separate branches (OTU1) (Figure 5).

The *T*. *subterranea* Ku-5^T^ strain was previously isolated from the water of the studied well [6]. Three of the OTUs obtained (OTU10, OTU6, OTU1) can be related to *T*. *subterranea* Ku-5^T^ based on its homology with *T*. *subterranea* (95.35, 99.67, 97.01%, respectively). OTE6 probably represents a strain very close to Ku-5. The analysis also showed that the *T*. *subterranea* phylotypes DNT52, DNT53, H33 are most likely present in the studied mat because OTU10 has 100% identity with them. The group of *T*. *subterranea* strains DNT52, DNT53, H33, and *T*. *subterranea* Ku-5^T^ have an ANI value of 94.6%, close to the species threshold (95%), which does not allow them to be identified as a separate species within the genus *Thiothrix*, while the *tilS* gene sequences of these strains are identical. However, there is a difference in the set of genes in terms of dissimilatory nitrogen metabolism. The *T*. *subterranea* strains DNT52, DNT53, and H33, unlike *T*. *subterranea* Ku-5^T^, have the *nirS* and *cnorBC* genes, which reduce nitrite to nitric oxide and nitric oxide to nitrous oxide in a stepwise manner [Unpublished data]. The homology of *tilS* OTU1 with *T*. *subterranea* DNT52, DNT53, and H33 is 92.36%, and with *T*. *subterranea* Ku-5^T^, it is 97.01%. On the phylogenetic tree, this phylotype is separate from the other *T*. *subterranea* strains. Thus, the species of *T*. *subterranea* in the obtained sample is represented by OTU10 and OTU6 and, probably, by OTU1.

On the phylogenetic tree, OTU8 and OTU3 are clustered with *T*. *lacustris* and *T*. *litoralis* species. Unfortunately, the *tilS* gene is not applicable for their separation. However, on the basis of *tilS* homology, it can be inferred that both of these species are present in the studied bacterial mat. The OTU8 has 99.67% homology with *T*. *litoralis* AS and 98.01% homology with *T*. *lacustris* BL^T^. In contrast, OTE3 has 98.01% homology with *T*. *lacustris* BL^T^ and 97.01% homology with *T*. *litoralis* AS^T^. Even closer to OTE3 is the *T*. *lacustris* strain WS (99.34% identity). The main difference between *T*. *lacustris* WS and *T*. *lacustris* BL^T^ is the presence of the *nif* gene cluster in the genome of the WS strain.

OTU2 probably belongs to a new species of *Thiothrix*. Its closest relative is *T*. *fructosivorans* Q^T^, but the homology of the *tilS* sequences is only 91.03%.

## 4. Conclusions

Recently, prokaryote phylogenomics based on the use of full genome sequences has found answers to many questions. Databases have been expanded with the genomes of uncultivated and difficult-to-cultivate bacteria. However, obtaining full genome sequences, especially when analyzing the metagenomes of microbial communities, is difficult in some cases. Although the 16S rRNA gene is a universal phylogenetic marker, it does not always allow for the determination of the taxonomic position and the identification of closely related species. The search for new genes that can substitute the use of 16S rRNA without losing reliability and informativeness is one of the developing fields in microbiology.

The problems of the phylogeny of the genus *Thiothrix* depend on the poor performance of the 16S rRNA gene with respect to the determination of the taxonomic relationships between closely related species.

Recently, based on whole genome comparisons, the ANI, AAI, and dDDH indices have been used to establish the exact taxonomic position of new isolates of the genus *Thiothrix*. However, the need for the sequencing of a large number of isolates with unclear taxonomic identity or the inability to assemble MAG’s from natural environments hindered the study of phylogenetic diversity of the genus *Thiothrix*. Therefore, the identification of genes that can clarify the systematics of the genus will facilitate phylogenetic studies of the genus *Thiothrix*. As a result of the evaluation of the bacterial core gene set, the genes *tilS* and *rpoB* were selected as potential phylogenetic markers to determine the taxonomic affiliations of species within the genus *Thiothrix*. The revealed homology thresholds for the *tilS* and *rpoB* sequences for the separation of the species of the genus *Thiothrix* are 91.85% and 95.59%, respectively. In contrast to the 16S rRNA gene, the *tilS* and *rpoB* genes make it possible to determine the species affiliations of isolates and to estimate the species composition of *Thiothrix* in natural ecosystems without the need for full genome sequencing and the assembly of MAGs.

Simple and reliable tools for phylogenetic analyses are required both to construct a systematic of the genus and to study its ecology in natural and anthropogenic systems. Understanding the species composition of *Thiothrix* is likely to help control its abundance where its abundant fouling is undesirable.

## Figures and Tables

**Table 1:** The general properties of the assembled *Thiothrix* genomes that were used for pangenome analysis.

Species	GenBank Accession	Genome Size (bp)	Contigs	G + C Content (mol%)
*T*. *subterranea* H33	JAVFKN000000000	4,297,497	124	51.4
*T*. *subterranea* DNT53	CP133197	4,204,657	1	51.3
*T*. *subterranea* DNT52	CP133216, CP133217	4,407,357	2	51.4
*T*. *lacustris* MK1	CP133218, CP133219, CP133220	3,647,642	3	51.2
*T*. *lacustris* WS	CP133333, CP133334, CP133335	4,117,441	3	51.4

## Data Availability

MAGs of *Thiothrix* spp. have been deposited in NCBI GenBank database under the accession numbers JAVFKN000000000 for *T. subterranea* H33; CP133197 for *T. subterranea* DNT53; CP133216, CP133217 for *T. subterranea* DNT52; CP133218, CP133219, CP133220 for *T. lacustris* MK1; CP133333, CP133334, CP133335 for *T. lacustris* WS.

## Supplementary Materials

The following supporting information can be downloaded at: https://www.mdpi.com/article/10.3390/microorganisms11102521/s1, Figure S1: Phylogenetic trees of the genus *Thiothrix* based on part nucleotide sequences of *tilS* (A) and *rpoB* (B) genes. The trees are drawn based on the maximum likelihood. The numbers at branch nodes are bootstrap values (expressed as percentages of 1000 replicates).

## References

[1] Grabovich M.Y., Ravin N.V., Boden R. (2023). Thiothrix. Bergey’s Manual of Systematics of Archaea and Bacteria.

[2] Ravin N.V., Rossetti S., Beletsky A.V., Kadnikov V.V., Rudenko T.S., Smolyakov D.D., Moskvitina M.I., Gureeva M.V., Mardanov A.V., Grabovich M.Y. (2022). Two new species of filamentous sulfur bacteria of the genus *Thiothrix*, *Thiothrix winogradskyi* sp. nov. and ‘*Candidatus* Thiothrix sulfatifontis’ sp. nov. Microorganisms.

[3] Rabenhorst G.L. (1865). Flora Europaea Algarum Aquae Dulcis et Submarinae.

[4] Winogradsky S. (1888). Beiträge zur morphologie und physiologie der bacterien. Heft I. Zur Morphologie UND Physiologie der Schwefelbacterien.

[5] Larkin J.M., Shinabarger D.L. (1983). Characterization of *Thiothrix nivea*. Int. J. Syst. Bacteriol..

[6] Ravin N.V., Rudenko T.S., Smolyakov D.D., Beletsky A.V., Rakitin A.L., Markov N.D., Fomenkov A., Sun L., Roberts R.J., Novikov A.A. (2021). Comparative genome analysis of the genus *Thiothrix* involving three novel species, *Thiothrix subterranea* sp. nov. Ku-5, *Thiothrix litoralis* sp. nov. AS and “*Candidatus* Thiothrix anitrata” sp. nov. A52, revealed the conservation of the pathways of dissimilatory sulfur metabolism and variations in the genetic inventory for nitrogen metabolism and autotrophic carbon fixation. Front. Microbiol..

[7] Stackebrandt E., Goebel B.M. (1994). Taxonomic note: A place for DNA-DNA reassociation and 16S rRNA sequence analysis in the present species definition in bacteriology. Int. J. Syst. Evol. Microbiol..

[8] Chernousova E., Gridneva E., Grabovich M., Dubinina G., Akimov V., Rossetti S., Kuever J. (2009). *Thiothrix caldifontis* sp. nov. and *Thiothrix lacustris* sp. nov., gammaproteobacteria isolated from sulfide springs. Int. J. Syst. Evol. Microbiol..

[9] Boden R., Scott K.M. (2018). Evaluation of the genus *Thiothrix* Winogradsky 1888 (Approved Lists 1980) emend. Aruga et al. 2002: Reclassification of *Thiothrix disciformis* to *Thiolinea disciformis* gen. nov., comb. nov., and of *Thiothrix flexilis* to *Thiofilum flexile* gen. nov., comb nov., with emended description of *Thiothrix*. Int. J. Syst. Evol. Microbiol..

[10] Howarth R., Unz R.F., Seviour E.M., Seviour R.J., Blackall L.L., Pickup R.W., Jones J.G., Yaguchi J., Head I.M. (1999). Phylogenetic relationships of filamentous sulfur bacteria (*Thiothrix* spp. and Eikelboom type 021N bacteria) isolated from wastewater-treatment plants and description of *Thiothrix eikelboomii* sp. nov., *Thiothrix unzii* sp. nov., *Thiothrix fructosivorans* sp. nov. and *Thiothrix defluvii* sp. nov. Int. J. Syst. Bacteriol..

[11] Rossetti S., Blackall L.L., Levantesi C., Uccelletti D., Tandoi V. (2003). Phylogenetic and physiological characterization of a heterotrophic, chemolithoautotrophic *Thiothrix* strain isolated from activated sludge. Int. J. Syst. Evol. Microbiol..

[12] Mekadim C., Killer J., Mrázek J., Bunešová V., Pechar R., Hroncová Z., Vlková E. (2018). Evaluation of the *infB* and *rpsB* gene fragments as genetic markers intended for identification and phylogenetic analysis of particular representatives of the order *Lactobacillales*. Arch. Microbiol..

[13] Mekadim C., Killer J., Pechar R., Mrázek J. (2018). Variable regions of the *glyS*, *infB* and *rplB* genes usable as novel genetic markers for identification and phylogenetic purposes of genera belonging to the family *Propionibacteriaceae*. Int. J. Syst. Evol. Microbiol..

[14] Táncsics A., Benedek T., Szoboszlay S., Veres P.G., Farkas M., Máthé I., Márialigeti K., Kukolya J., Lányi S., Kriszt B. (2015). The detection and phylogenetic analysis of the alkane 1-monooxygenase gene of members of the genus *Rhodococcus*. Syst. Appl. Microbiol..

[15] Edgar R.C. (2010). Search and clustering orders of magnitude faster than BLAST. J. Bioinform..

[16] Guindon S., Gascuel O. (2003). A simple, fast, and accurate algorithm to estimate large phylogenies by maximum likelihood. Syst. Biol..

[17] Anisimova M., Gascuel O. (2006). Approximate likelihood ratio test for branches: A fast, accurate and powerful alternative. Syst. Biol..

[18] Bankevich A., Nurk S., Antipov D., Gurevich A.A., Dvorkin M., Kulikov A.S., Lesin V.M., Nikolenko S.I., Pham S., Prjibelski A.D. (2012). SPAdes: A new genome assembly algorithm and its applications to single-cell sequencing. J. Comput. Biol..

[19] Kolmogorov M., Yuan J., Lin Y., Pevzner P.A. (2019). Assembly of long, error-prone reads using repeat graphs. Nat. Biotechnol..

[20] Walker B.J., Abeel T., Shea T., Priest M., Abouelliel A., Sakthikumar S., Cuomo C.A., Zeng Q., Wortman J., Young S.K. (2014). Pilon: An integrated tool for comprehensive microbial variant detection and genome assembly improvement. PLoS ONE.

[21] Chaumeil P.A., Mussig A.J., Hugenholtz P., Parks D.H. (2019). GTDB-Tk: A toolkit to classify genomes with the Genome Taxonomy Database. J. Bioinform..

[22] Guindon S., Dufayard J.F., Lefort V., Anisimova M., Hordijk W., Gascuel O. (2010). New algorithms and methods to estimate maximum-likelihood phylogenies: Assessing the performance of PhyML 3.0. Syst. Biol..

[23] Lee I., Ouk Kim Y., Park S.C., Chun J. (2016). OrthoANI: An improved algorithm and software for calculating average nucleotide identity. Int. J. Syst. Evol. Microbiol..

[24] Yarza P., Yilmaz P., Pruesse E., Glöckner F.O., Ludwig W., Schleifer K.H., Whitman W.B., Euzéby J., Amann R., Rosselló-Móra R. (2014). Uniting the classification of cultured and uncultured bacteria and archaea using 16S rRNA gene sequences. Nat. Rev. Microbiol..

[25] Konstantinidis K.T., Tiedje J.M. (2005). Towards a genome-based taxonomy for prokaryotes. J. Bacteriol..

[26] Jain C., Rodriguez-R L.M., Phillippy A.M., Konstantinidis K.T., Aluru S. (2018). High throughput ANI analysis of 90K prokaryotic genomes reveals clear species boundaries. Nat. Commun..

[27] Na S.I., Kim Y.O., Yoon S.H., Ha S.M., Baek I., Chun J. (2018). UBCG: Up-to-date bacterial core gene set and pipeline for phylogenomic tree reconstruction. J. Microbiol..

[28] Kim J., Na S.I., Kim D., Chun J. (2021). UBCG2: Up-to-date bacterial core genes and pipeline for phylogenomic analysis. J. Microbiol..

[29] Fabret C., Dervyn E., Dalmais B., Guillot A., Marck C., Grosjean H., Noirot P. (2011). Life without the essential bacterial tRNA Ile2-lysidine synthetase TilS: A case of tRNA gene recruitment in Bacillus subtilis. Mol. Microbiol..

[30] Panova I.A., Rusanov I.I., Kadnikov V.V., Latygolets E.A., Avakyan M.R., Ivanov M.V., Zyusman V.C., Kovaleva A.A., Ravin N.V., Pimenov N.V. (2020). Sulfate reduction in underground horizons of a flooded coal mine in Kuzbass. Microbiology.

