# Supplementary material for: tilS and rpoB: New Molecular Markers for Phylogenetic and Biodiversity Studies of the Genus Thiothrix
Source: Microorganisms. 2023 Oct 9;11(10):2521. doi: 10.3390/microorganisms11102521 (PMC10609254; doi:10.3390/microorganisms11102521)
Supplement: Supplementary file 1 [file microorganisms-11-02521-s001.zip › microorganisms-2607911-supplementary.pdf]

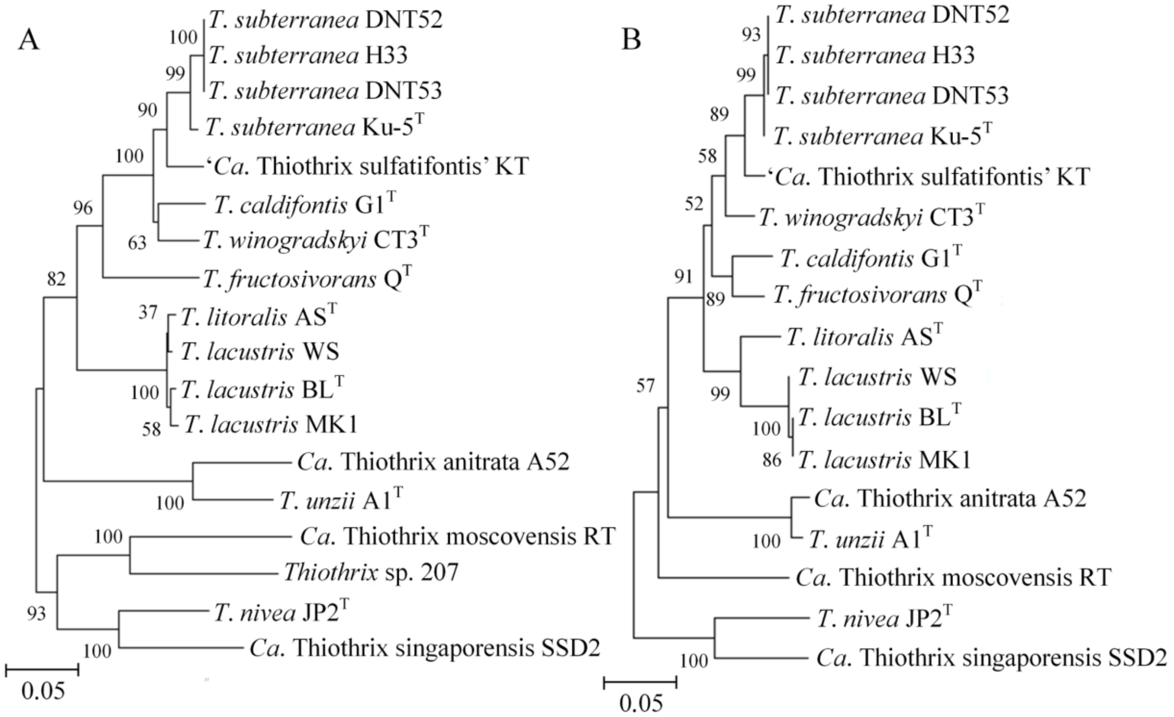

Figure S1. Phylogenetic trees of the genus *Thiothrix* based on part nucleotide sequences of *tilS* (A) and *rpoB* (B) genes. The trees are drawn based on the maximum likelihood. The numbers at branch nodes are bootstrap values (expressed as percentages of 1000 replicates).
